# Cyclodextrin-poly(ε-caprolactone) based nanoparticles able to complex phenolphthalein and adamantyl carboxylate

**DOI:** 10.3762/bjnano.5.76

**Published:** 2014-05-16

**Authors:** Daniela Ailincai, Helmut Ritter

**Affiliations:** 1 Centre of Advanced Research in Bionanoconjugates and Biopolymers, “Petru Poni” Institute of Macromolecular Chemistry of Romanian Academy, 41A Aleea Grigore Ghica Voda, 700487 Iasi, Romania; 2 Heinrich-Heine-Universität, Institut für Organische Chemie und Makromolekulare Chemie, Geb. 26.33, Eb. 00, Universitätsstr. 1, 40225 Düsseldorf, Germany

**Keywords:** click reaction, complexation, cyclodextrin, encapsulation, nanoparticles

## Abstract

A new compound composed of poly(ε-caprolactone) and β-cyclodextrin (β-CD) was synthesized by click chemistry. This compound was used to obtain stable nanoparticles, which have been proven to be able to complex phenolphthalein and adamantyl carboxylate. The nanoparticles are characterized by a distinct morphology, i.e., a hydrophobic core formed by the polyester chain and a shell containing the CD part. Moreover, the formed nanoparticles have been proven to encapsulate umbelliferone in the polyester phase, which may serve as an example for the uptake of a drug. The formed nanoparticles were characterized in terms of sizes and morphology by both DLS and TEM.

## Introduction

The development of new nanoscale systems for the encapsulation of drugs or imaging agents, which could be used in the treatment, localization or diagnosis of diseased tissues, represents one of the most interesting aims for researchers working in the realm of biochemistry. A lot of such systems have been reported in the past years, including nanovesicles based on natural macromolecular compounds, liposomes formed by autoassemble of phospholipids in aqueous medium [1], and nanovesicles formed by the autoassemble of synthetic amphiphilic copolymers, polymersomes [2]. There is a broad variety of conditions which have to be met, both for nanoparticles and for the macromolecular compounds that underlie their formation [3].

The existence of various barriers present at different levels of the human body, necessitates the use of nano-level carriers. Regarding the requirements for the macromolecular compounds used to form nanoparticles, among the most important are biocompatibility, biodegradability and nontoxicity [4–5]. The aliphatic polyesters combine the requirements above mentioned. Consequently, they had a huge impact on the biomedical field and are used in surgical sutures, tissue scaffolding and for bone screws [6]. Between them, one of the most studied and used is poly(ε-caprolactone), a linear aliphatic polyester, obtained by ring-opening polymerization of ε-caprolactone.

Poly(ε-caprolactone) was used in several combinations, including click reaction products. Functionalized polyester was obtained, which was further used for grafting β-CD on it. The final product has been proven to exhibit a host–guest ability with different compounds [7]. Pseudo-polyrotaxanes based on propargyl functionalized poly(ε-caprolactone) and mono-(6-azido-6-desoxy)-ß-CD have been successfully obtained [8].

Here, we propose to obtain an amphiphilic behavior by using poly(ε-caprolactone), which is known for its high hydrophobicity. To obtain an amphiphilic behavior requires the presence of a hydrophilic compound, which also has to comply with the biocompatibility, biodegradability and nontoxicity requirements. Copper catalyzed click chemistry became a very important tool for the synthesis of new polymeric structures over the past years [9] and represents an attractive method for bonding a polyester chain with a hydrophilic compound [10].

The use of propargylic alcohol as an initiator leads to a polyester with an acetylenic end group, which allows its use as a click reaction precursor. The chemical modification of CD, consisting of the selective substitution of the C6-hydrogen with an azidic group, permits its use as a click reaction partner for the modified poly(ε-caprolactone). CDs are cyclic oligosaccharides composed of α-(1-4)-linked α-D-glucosyl units [11]. They have a hydrophobic cavity in their interior, whereas the exterior part is hydrophilic. Drugs with adequate sizes can be complexed by the internal hydrophobic part of the CDs, while macromolecular drugs are only partially included [12]. Host–guest interactions between a monoacrylated CD and *N*-isopropylacrylamide based copolymer have been reported [13]. The characterization and the morphology of a complex based on fish oil encapsulated in CD with the use of polycaprolactone have also been reported [14–15]. Furthermore, CD inclusion complexes with different essential oils and their potential application for antimicrobial delivery have been described [16].

The aim of this study was to develop a β-CD-poly-ε-caprolactone compound by click chemistry, which is able to form nanoparticles in water. Once obtained, the nanoparticles were used for host–guest behavior studies with different hydrophobic compounds, including phenolphthalein and adamantyl carboxylate. The ability of the click reaction product to complex umbelliferone was also investigated, based on its structural similarity with other hydrophobic compounds previously described in the literature as guests for CD.

## Experimental

### Materials, general remarks

β-CD was obtained from Wacker Chemie GmbH, Burghausen, Germany and was used after being dried overnight with a vacuum oil pump over P4O10. *N*,*N*-Dimethylformamide (DMF) was purchased from Fluka, Germany. Dimethyl sulfoxide-*d*_6_ (99.9 atom % D) and deuterium oxide. ε-Caprolactone was purchased from Acros, dried over calcium hydride, distilled under reduced pressure, and stored over 0.4 nm molecular sieves. Sodium ascorbate (AppliChem) and copper(II) sulfate (Carl Roth GmbH) were used as received. Commercially available reagents and solvents were used without further purification.

#### Measurements

^1^H NMR spectra were recorded on a Bruker Avance DRX 600 at 20 °C by using DMSO-*d*_6_ or CDCl_3_ (99.9%) as solvents. Chemical shifts referenced to the solvent value δ = 2.5 ppm for DMSO-*d*_6_ and δ = 7.26 ppm for CDCl_3_. SEC-MALS measurements were carried out on a combined system comprising the following elements: refractive-index detector Optilabrex (Wyatt Technologies, laser wavelength 658 nm), three-angle light-scattering detector miniDawn TREOS (Wyatt Technologies, laser wavelength 658 nm, detector angles at 43.5°, 90.0° and 136.5°), UV detector Waters 486 (Waters), column set of HEMAbio 300 and HEMAbio 100 (MZ-Analysentechnik), pump, degasser and autosampler (Agilent 1200, Agilent technologies). The eluent was ultrapure water at a flow rate of 1 mL/min. The molecular weight was calculated with Astra5 software from static-light-scattering data by using the Zimm model. Dynamic light scattering (DLS) experiments were carried out with a Malvern Zetasizer Nano ZS ZEN 3600 at a temperature of 25 °C. The particle size distribution is derived from a deconvolution of the measured intensity autocorrelation function of the sample by a general purpose method, i.e., the non-negative least squares algorithm, included in the DTS software. The TEM images have been obtained with a TEM Zeiss 902.

### Synthesis

#### Synthesis of alkyne-functionalized polyester

The strategy for the synthesis of functionalized aliphatic polyester bearing a propargyl end-group relies on the Sn(Oct)-mediated ring-opening polymerization (ROP) of ε-caprolactone by using the propargylic alcohol as an initiator. A 10 mL round bottom flask was heated and purged with argon. 5 g (43.806 mmol, 10 Eg) of ε-caprolactone and 608.86 mg of propargylic alcohol was added to the round-bottom flask and heated at 120 °C. When the temperature is reached, 88.72 mg (0.219 mmol) of Sn(Oct)_2_, 0.5 mol % with respect to the monomer, is added. Argon was purged into the reaction flask. The reaction mixture was stirred for 24 h. After this, the product was cooled at room temperature and a solid is formed. The product was dissolved in CH_2_Cl_2_, separated through precipitation in a large excess of hexane, and dried under vacuum (91% mass yield). The reaction product was characterized in terms of molecular weight and dispersity by GPC and the obtained values were 4600 for the Mw and 1,164 for the dispersity index. The structure was proved by NMR. ^1^H NMR (600 MHz, CDCl_3_): 1.15 (H from acetylenic group), 1.3 (H-e), 1.6 (H-d), 2.3 (H-c), 3.6 (H-g'), 4 (H-g).

#### Synthesis of the CD endgroup containing poly(ε-caprolactone) by a “click” reaction

Mono-(6-azido-6-desoxy)-ß-CD has been obtained following the published procedure [7]. In a similar manner as described before [8] the click reaction was successfully realized by adding mono-(6-azido-6-desoxy)-ß-CD to a solution of polyester in DMF (0.6% w/w). To the reaction mixture were added 10 mol % sodium ascorbate and 5 mol % copper(II) sulfate pentahydrate. The flask was immersed in an oil bath at 95 °C for 24 h. The solvent was removed at the rotatory evaporator and the product was dried under vacuum.

^1^H NMR (600 MHz, C_2_D_6_SO) 3.32 (H-2,4), 3.6–3.84 (H-3,5,6), 4.44–4.6 (OH-6), 4.85 (H-1), 5.73 (OH-2, 3) from the β-CD rest and 1.15 (H), 1.3 (H-e), 1.6 (H-d), 2.3 (H-c), 3.6 (H-g'), 4 (H-g) from the polyester. The melting point was 77 °C.

**Figure 1:**
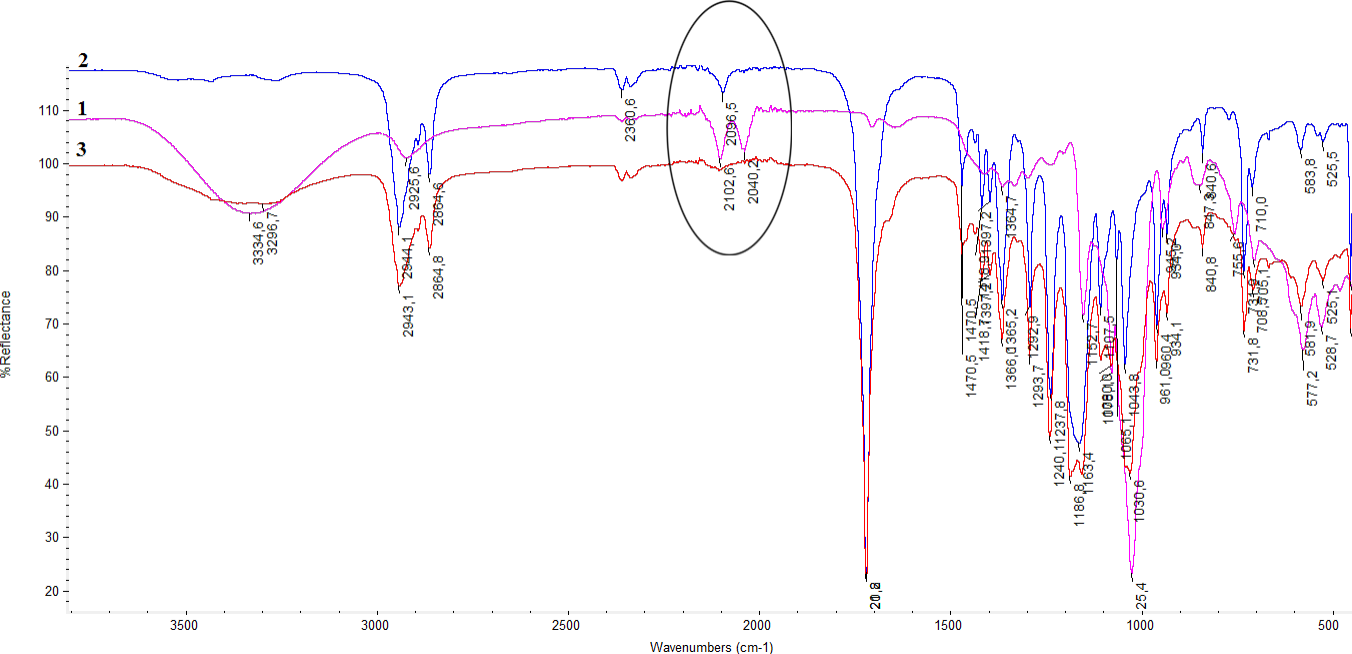
The FTIR spectra for (1) the mono-6-azido-CD, (2) the alkyne compound of poly(ε-caprolactone), and (3) the click reaction product.

The success of the click reaction between the poly(ε-caprolactone) with an acetylenic final group and the modified β-CD was also proved by FTIR analysis. The polyester presented one peak at 2096 cm^−1^ corresponding to the acetylenic group, while the modified β-CD presented two peaks, one at 2040 cm^−1^ and one at 2100 cm^−1^, corresponding to the azidic group. The click reaction product had no peaks in these region since all reactive groups (acetylenic and azidic) have been consumed during the reaction (Figure 1).

#### Preparation of nanoparticles

A solution of the click reaction compound (Figure 1, (3)) was prepared by dissolving 100 mg compound into 1 mL of DMF. Small amounts from this solution were added to a large excess of water (35 mL) under an ultrasonifier treatment. The formation of aggregates was proved by means of DLS measurements.

#### Phenolphthalein and adamantyl carboxylate complexation by the formed nanoparticles

A suspension of the nanoparticles (A) was obtained as previously described. A solution of phenolphthalein in water was prepared, to which trace amounts of NaOH were added to increase the pH and obtain a pinkish solution (B). A was added to B, while stirring (C). To C, a solution of adamantly-carboxylate in water was added, while vigorously stirring.

## Results and Discussion

**Scheme 1:**
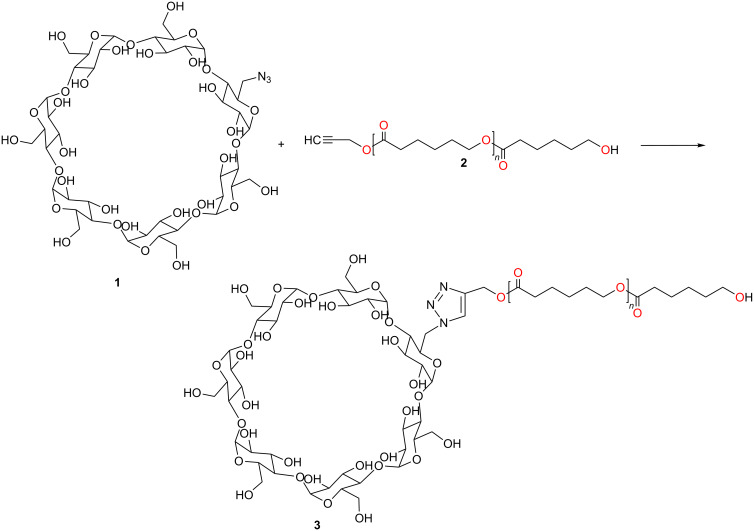
Click reaction between mono-6-azido-CD (**1**) and the alkyne compound of poly(ε-caprolactone) (**2**).

Previously synthesized mono-6-azido-β-CD (**1**) was “clicked” with an alkyne compound of poly(ε-caprolactone) (**2**) (Scheme 1). The reaction was catalyzed by copper(I), obtained in situ by the reduction of CuSO_4_ with sodium ascorbate. DMF was used as a solvent due its high boiling point and its ability to solubilize both starting materials. The click reaction product was characterized by using ^1^H NMR and FTIR spectroscopy.

**Figure 2:**
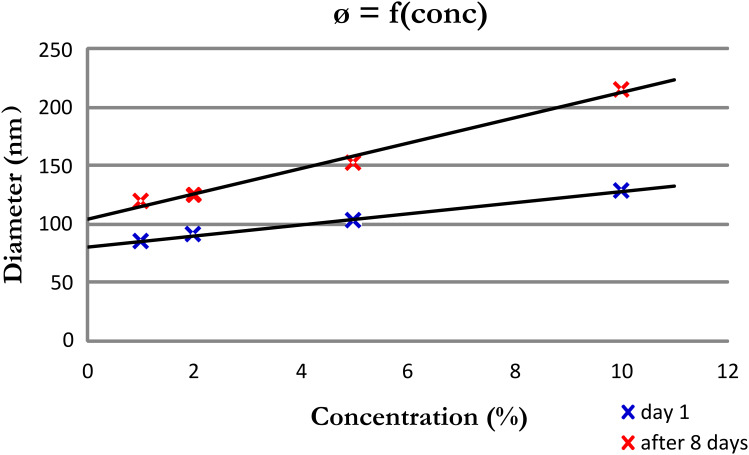
The dependency between the nanoparticles’ diameter (formed by **3** in water) as a function of the concentration.

The properties of **3** in water were investigated by DLS measurements. We observed that the higher the concentration of the copolymer the greater the sizes of the nano-aggregates. These nano-aggregates are quite stable in water as DLS measurements of the suspension, performed after 8 days, shows an increase of the nano-aggregates’ diameter of ≈35% (Figure 2).

**Figure 3:**
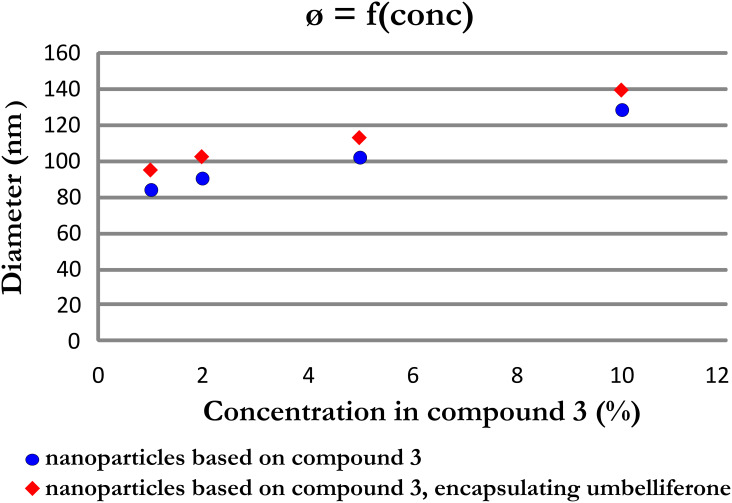
Diameter of the nanoparticles based on compound **3**, with and without umbelliferone.

The nanoparticles were prepared by adding small amounts of a solution of **3** in DMF to a solution of umbelliferone in ultrapure water (35 mL). The amount of the added umbelliferone was varied in this experimental protocol. We observed that the diameter of the nanoparticles increased with an increasing umbelliferone concentration (Figure 3). Moreover, the diameter of the nanoparticles encapsulating umbelliferone was bigger than the diameter of the nanoparticles without umbelliferone. The increase of the diameter of the nanoparticles formed in the umbelliferone solution can be explained by the entrapment of umbelliferone, a highly hydrophobic compound, in the hydrophobic core of the nanoparticles.

**Figure 4:**
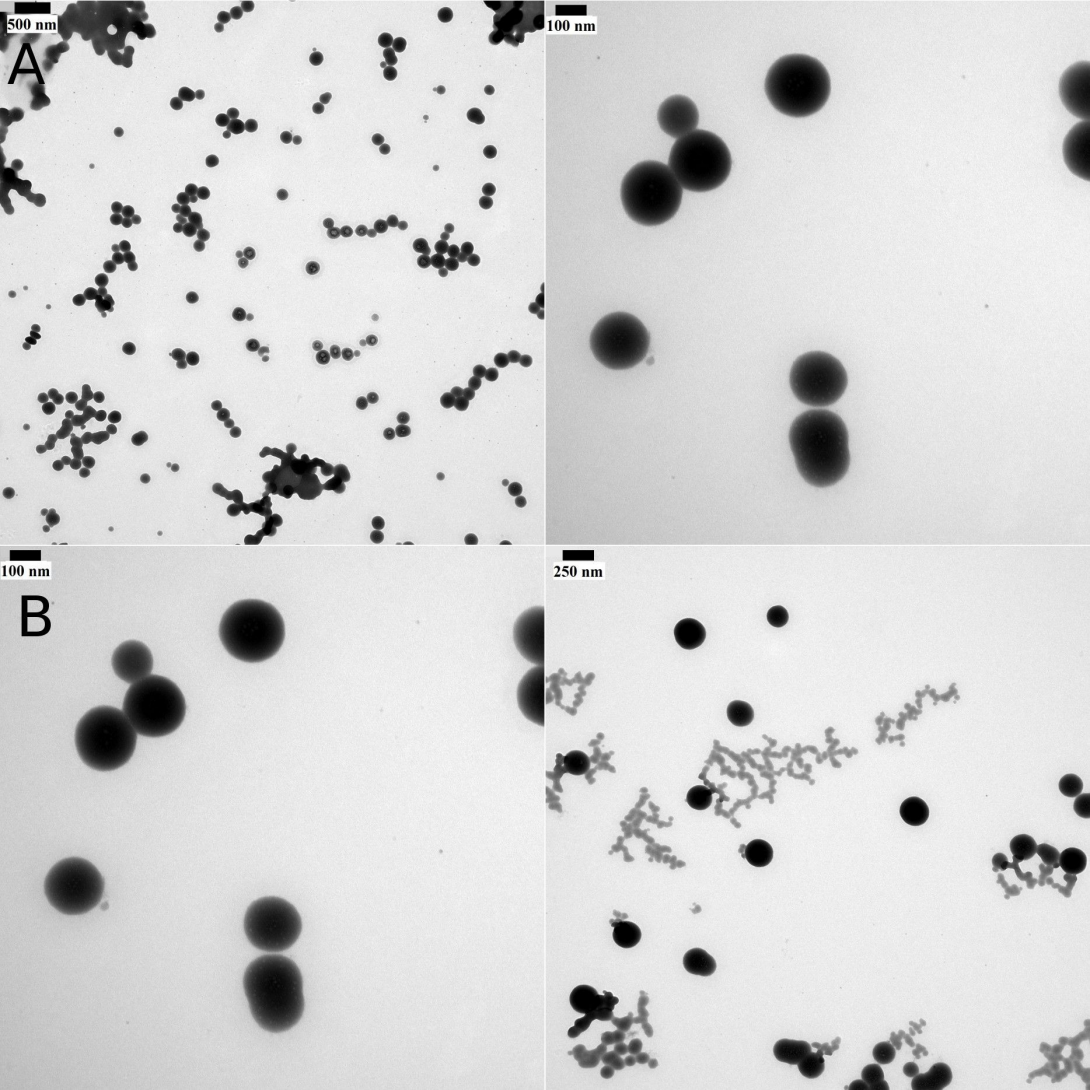
TEM images for the nanoparticles based on **3**. The nanoparticles were obtained by dispersing the click reaction product (**3**) in water. with a concentration of 1% (A) and 2% (B).

To investigate the morphology of the formed nanoaggregates, TEM analysis was performed. The microscope images show the formation of nanoparticles the sizes of which are in agreement with the DLS measurements. TEM images of nanoparticles obtained with two different concentrations are presented in Figure 4 (A: concentration 1%, B: concentration 2%).

**Figure 5:**
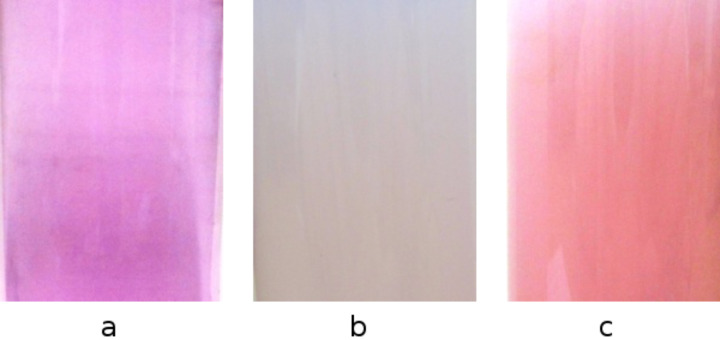
The color during the complexation with phenolphthalein and adamantyl carboxylate. (a) Phenolphthalein solution in water at pH > 7, (b) nanoparticles suspension added to the phenolphthalein solution, (c) nanoparticles suspension in phenolphthalein solution after the addition of adamantyl carboxylate.

We also tested the ability of the formed nanoparticles to complex phenolphthalein and adamantyl carboxylate. Furthermore, this study also aimed for establishing the exact morphology of the nanoparticles. Pictures were taken of the phenolphthalein solution in water at pH > 7, the nanoparticles suspension added to the phenolphthalein solution, and the adamantyl carboxylate added to the previous phase (Figure 5).

**Figure 6:**
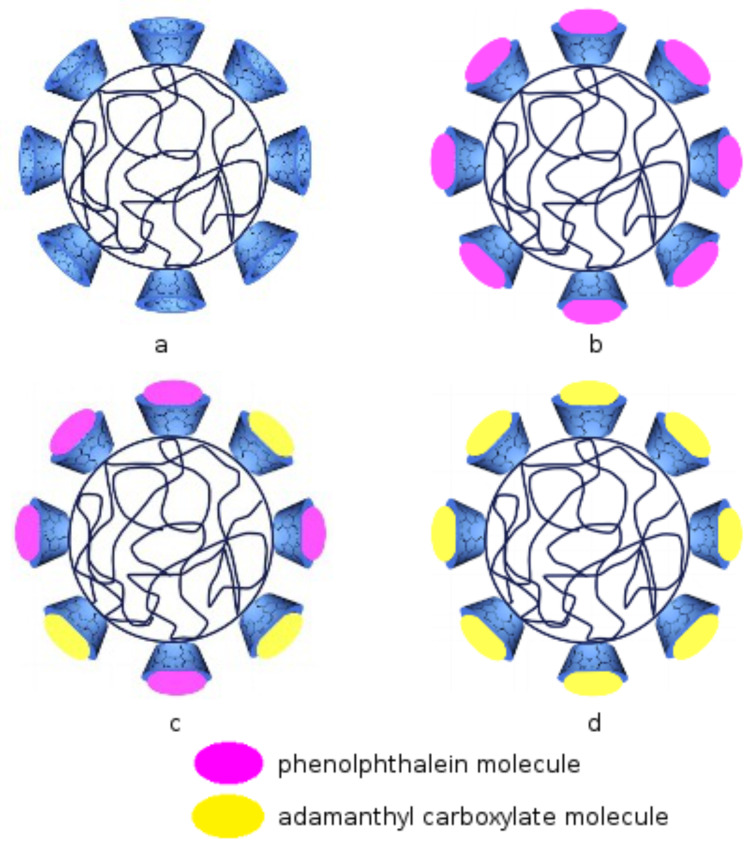
Schematic representation of the CD based nanoparticles morphology. (a) In water at pH > 7, (b) in phenolphthalein solution, (c) after small amounts of adamantyl carboxylate were added to the phenolphthalein solution, (d) after a large amount of adamantyl carboxylate was added to the phenolphthalein solution.

Based on the ability of the nanoparticles to complex phenolphthalein and adamantyl carboxylate we propose the morphology shown in Figure 6.

Presumably, the interior is formed by the hydrophobic poly(ε-caprolactone) chain, while the β-CD part is located on the exterior (Figure 6a). When the nanoparticles suspension is added to the phenolphthalein solution (pH > 7) the phenolphthalein is included in the hydrophobic cavity of β-cyclodextrin (Figure 6b) and the suspension changes its color from pink in white (Figure 5). The affinity of adamantyl carboxylate to β-cyclodextrin is higher than of phenolphthalein to β-cyclodextrin. Thus, the addition of adamantyl carboxylate results in phenolphthalein leaving the β-cyclodextrin cavity and adamantyl carboxylate entering it so that the suspension changes its color back to pink (Figure 5).

## Conclusion

In conclusion, we successfully prepared nanoparticles in water by nano-precipitation of a solution of poly(ε-caprolactone) tailored with the addition of β-CD as an endgroup. We proved that the formed nanoparticles are able to complex phenolphthalein and adamantyl carboxylate, respectively, due to their distinct morphology. They consist of a hydrophobic core composed of the polyester chain and a shell, to which the β-CD part is covalently attached. The nanoparticles have been characterized in terms of morphology and diameter by both DLS measurements and TEM. Umbelliferone, a hydrophobic molecule, was not included in the cavity of the CD moiety according to ROESY NMR measurements. However, the encapsulation of umbelliferone in the hydrophobic part of the nanoparticles was demonstrated by the increased diameter of nanoparticles obtained in a solution with umbelliferone compared to nanoparticles obtained in pure water.
